# Risk factors associated with hospital transfer among mild or asymptomatic COVID-19 patients in isolation facilities in Tokyo: a case-control study

**DOI:** 10.1016/j.ijregi.2021.11.001

**Published:** 2021-11-17

**Authors:** Keisuke Naito, Tomoyo Narita, Yukari Murata, Naoto Morimura

**Affiliations:** a Bureau of Social Welfare and Public Health, Tokyo Metropolitan Government, 8-1 Nishi-Shinjuku 2-chome, Shinjuku-ku, Tokyo, Japan; b Tokyo Metropolitan Institute of Public Health, 3-24-1 Hyakunin-cho, Shinjuku-ku, Tokyo, Japan; c Department of Emergency Medicine, Teikyo University School of Medicine, 2-11-1 Kaga, Itabashi-Ku, Tokyo, Japan

**Keywords:** COVID-19, Case-control, Multivariable analysis, Prehospital, Telemedicine, Tokyo

## Abstract

**Objectives:**

The Tokyo Metropolitan Government has been implementing facility-based isolation of asymptomatic/mild coronavirus disease (COVID-19) patients to facilitate timely hospital referral. However, there are only a few published studies in prehospital settings, and the factors associated with hospital transfer are unclear. Our study identified the factors associated with COVID-19 deterioration in a prehospital setting.

**Methods:**

This case-control study assessed the risk factors for hospital transfer from isolation facilities and the need for ambulance transport due to deterioration among COVID-19 patients, using multivariate logistic regression analysis.

**Results:**

In total, 10 590 patients (median age 34 years), with male predominance (61.1%), were included. 367 (3.5%) were transferred to hospital, of whom 44 (12.0%) required ambulance transport. Hypertension, diabetes, and bronchial asthma were prevalent in 704 (6.6%), 195 (1.8%), and 305 (2.9%) patients, respectively. After adjustment, older age, male sex, higher body mass index (BMI), and comorbidities (including diabetes, inflammatory bowel disease, and bronchial asthma) were associated with hospital transfer. Older age, male sex, and higher BMI significantly increased the risk of transfer by ambulance.

**Conclusions:**

Our results may be beneficial for the development of intervention measures for probable future COVID-19 waves.

## Highlights

• Facility-based isolation for COVID-19 in Tokyo involved remote health observation.
• This study was conducted in a prehospital setting using multivariable analysis.
• Older age, male sex, and higher BMI were associated with transfers to hospital.
• Comorbidities, such as bronchial asthma, increased the risk for hospital transfer.
• Some patients requiring urgent oxygenation showed few signs of dyspnea (silent hypoxia).

## Introduction

The coronavirus disease 2019 (COVID-19) pandemic has been a global public health crisis since the beginning of 2020, and continues to threaten the healthcare systems of many countries (World Health Organization 2021). Japan has experienced five waves of domestic infection to date, and more than 1.5 million people have been affected by the disease (Japanese Ministry of Health, Labour and Welfare, 2021; Saito et al., 2021). During the third wave, which started around November 2020, the number of daily reported cases surged drastically, peaking at 8045 on January 8, 2021 (Japanese Ministry of Health, Labour and Welfare, 2021).

Tokyo has been one of the epicenters of infection in the country, where a significant number of cases have been recorded over the five waves (Tokyo Metropolitan Government, 2021). Since April 2020, as with other Asian countries, the Tokyo Metropolitan Government has been implementing facility-based quarantine for asymptomatic or mildly symptomatic COVID-19 patients, to facilitate community isolation, frequent monitoring, triage, and referral (Chen et al., 2020a, Chen et al., 2020b, Chia et al., 2021, Her, 2020). Consequently, this has alleviated the shortage of medical resources and staff by suppressing the viral spread (Ranney et al., 2020; Emanuel et al., 2020; Chen et al., 2021; Wilasang et al., 2020; Pan et al., 2020; Cai et al., 2020; Dickens et al., 2020). However, during the third wave, hospitals, public health centers, and isolation facilities (repurposed hotel accommodation and non-health facilities) were overwhelmed by the high number of infected patients. The numbers of admissions to isolation facilities and hospital transfers, as well as patients presenting with more severe symptoms, such as hypoxemia, increased greatly. Subsequently, the role of isolation facilities in this public health crisis has become more significant. Therefore, it is of paramount importance to identify patients who are at risk of developing more severe symptoms and requiring medical attention.

An increasing number of clinical and epidemiological studies investigating the risk factors for COVID-19 deterioration have been published recently (Matsunaga et al., 2020; Popkin et al., 2020; Zheng et al., 2020; Yang et al., 2020; Liang et al., 2020; Myers et al., 2020; Brenner et al., 2020; Michelena et al., 2020; Hadi et al., 2020; Lippi and Henry, 2020; Fadini et al., 2020). However, only a few studies have been conducted at prehospital isolation facilities (Chia et al., 2021; Lee et al., 2020), and the factors associated with hospital transfer remain unknown.

Our aim was to conduct an epidemiological study among patients accommodated in prehospital isolation facilities in Tokyo, with hospital transfer because of worsening of COVID-19 symptoms being the primary outcome.

## Materials and methods

### Isolation facilities and health monitoring

**Figure 1:**
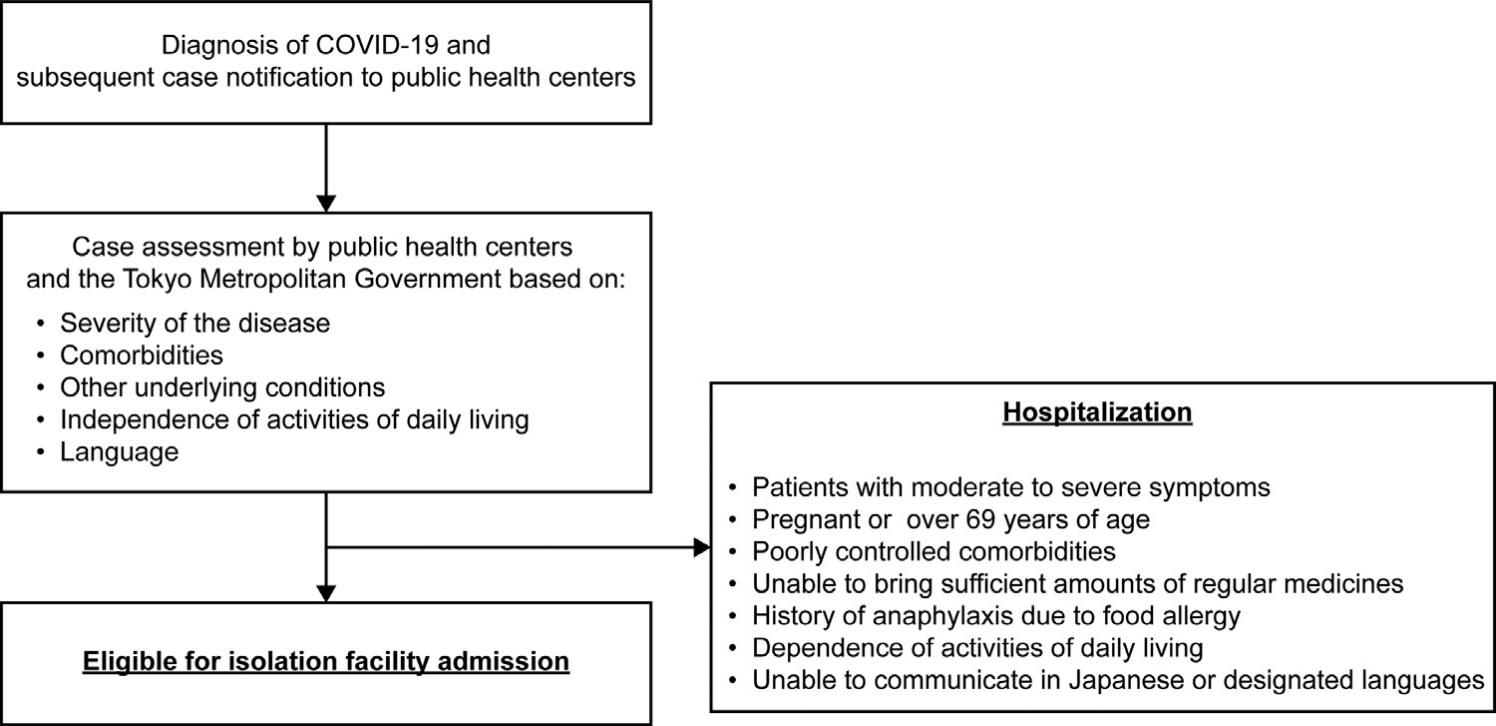
Eligibility criteria for admission to isolation facilities. A newly diagnosed case of COVID-19 is immediately reported to public health centers, whereafter clinical and epidemiological information is collected and subsequently sent to the Tokyo Metropolitan Government. Here physicians assess eligibility for admission to an isolation facility. Ineligible patients are hospitalized. Some cases requiring hospitalization are shown.

A flowchart for isolation facility admission eligibility is shown in Figure 1. Patients were mainly selected by public health centers and the Tokyo Metropolitan Government based on symptom severity, underlying conditions, and comorbidities.

During the study period, there were 11 isolation facilities in Tokyo, Japan. Patient information from nine of these, including facilities in Shinagawa, Toranomon, Asakusa, Ikebukuro, Kabukicho, Nishi-Shinjuku, Kiba, Minami-Tama, and Otsuka, was collected and analyzed. The period of isolation for symptomatic and COVID-19-positive patients was 10 days from symptom onset and 3 days from the resolution of fever and respiratory symptoms, whereas that for asymptomatic patients was 10 days following a positive test for COVID-19.

Health monitoring was mostly conducted remotely to minimize physical contact, except in emergencies. Patient data were recorded using an online-based health monitoring system called LAVITA® (Nihon Kohden, Tokyo, Japan). On admission, pulse oximeters and thermometers were allocated to each patient. Both patients and medical staff could enter vital signs (body temperature, SpO_2_, heart rate, etc.) and symptom data into LAVITA®. Most communication was through phone calls, although video calls were also available. This enabled physicians and nurses to make decisions based on visual and auditory information.

Patients could only be treated with commercially available drugs, such as antipyretics and oral rehydration solutions. Medical procedures could not be performed because the isolation facilities were not medical institutions. When medical tests and radiographic imaging were required by physicians, the patient would be subsequently transferred in either a depressurized car or by a private emergency service. If the physician recognized the need for urgent oxygen administration at the prehospital site, emergency medical services would be contacted, and the patient would then be transferred to the hospital by ambulance.

### Criteria for admission to isolation facilities

The criteria for admission to an isolation facility were laboratory-confirmed severe acute respiratory syndrome coronavirus 2, mild or asymptomatic disease, and not requiring hospitalization according to a physician assessment. Patients with moderate-to-severe symptoms, such as dyspnea, were not admitted to the isolation facilities; instead, they were hospitalized. Furthermore, pregnant patients and those aged > 65 years were hospitalized. It is important to note that the age restriction was modified around the end of 2020, allowing patients aged 65 to 69 years to be admitted to isolation facilities. Infectious patients who had already been treated and had recovered in hospital could also be transferred to the isolation facilities. In the event of poorly controlled underlying disease, patients were assessed by public health physicians, and hospitalization was arranged if the isolation facility was not adequately equipped to manage these cases. For instance, patients with a systolic blood pressure of > 180 mmHg or with HbA1c levels > 10% were rarely admitted to the isolation facilities. Therefore, only those with well-managed comorbidities were eligible for admission to the isolation facilities. Moreover, patients aged > 65 years with recognized risk factors, such as diabetes or chronic respiratory and cardiovascular diseases, were not admitted to the isolation facilities owing to high presumed risk, whereas those with hypertension were allowed because of its high prevalence.

### Study design and participants

This case-control study included patients who had been admitted to any of the nine isolation facilities in Tokyo from November 1, 2020 and discharged by January 31, 2021. The primary outcome was transfer from an isolation facility to a hospital because of worsening COVID-19 symptoms, and the secondary outcome was ambulance transport. These outcomes were assessed longitudinally during the study period.

The exclusion criteria were hospital discharge and asymptomatic infection. Patients who left the isolation facilities for other reasons, and those transferred to hospitals for reasons unrelated to COVID-19, were also excluded.

### Data collection

Patient information regarding age, sex, height, weight, underlying diseases, day of symptom onset, and viral testing was mainly obtained through initial interviews at the time of admission to the isolation facilities. As we could not directly access former clinical and hospital records, data regarding comorbidities, how these were controlled, and medications were mostly based on self-reporting. Health data after admission to the isolation facilities were collected from LAVITA®.

### Predictors of outcome

To identify the risk factors, predictors included age, sex, body mass index (BMI), and comorbidities, such as hypertension, chronic cardiovascular disease, chronic respiratory disease other than bronchial asthma, bronchial asthma, diabetes, dyslipidemia, hyperuricemia, chronic kidney disease, liver disease, recent use of immunosuppressive agents, history of stroke, history of malignancy, autoimmune collagen disease, inflammatory bowel disease, and human immunodeficiency virus. BMI was categorized into < 25, 25–30, and ≥ 30 kg/m^2^. Chronic respiratory diseases included chronic obstructive pulmonary disease and obstructive sleep apnea. Immunosuppressive agents included oral corticosteroids at any dose, immunosuppressants, and biologics. Almost all patients with a history of malignancy had been treated successfully and were devoid of active cancer. Patients with undiagnosed comorbidities and those only requiring follow-ups without treatment were excluded.

### Statistical analysis

Quantitative and categorical variables are presented as median (interquartile range [IQR]) and frequency (%), respectively. Statistical analyses were performed using the EZR software version 1.50, a statistical user interface for the R commander (Saitama Medical Center, Jichi Medical University, Saitama, Japan; R, version 3.6.3: The R Foundation for Statistical Computing, Vienna, Austria) (Kanda, 2013). All *p-*values were two-sided, and statistical significance was set at *p* < 0.05. The Mann–Whitney U, chi-square, and Fisher's exact tests, were used where appropriate.

Univariate and multivariate logistic regression models were used to determine the risks associated with hospital transfer. For the primary outcome, age, sex, BMI, and comorbidities, including hypertension, diabetes, cardiovascular, and chronic respiratory diseases, were included as independent variables, as these had been previously reported as risk factors (Matsunaga et al., 2020; Popkin et al., 2020; Zheng et al., 2020; Yang et al., 2020; Lippi and Henry, 2020; Fadini et al., 2020). Comorbidities with *p* < 0.20 in the univariate analysis were selected. For the secondary outcome, considering the rarity of the event and the small sample size (*n* = 44), variables selected to be included in the model were categorized by age, sex, and BMI.

### Ethics

This study was conducted in accordance with the ethical guidelines for medical and health research involving human subjects issued by the Japanese Ministry of Health, Labour and Welfare, as well as the Declaration of Helsinki. Informed consent was obtained by means of an opt-out method on the Tokyo Metropolitan Institute of Public Health website. The study protocol was approved by the ethics committee of the Tokyo Metropolitan Institute of Public Health.

## Results

**Figure 2:**
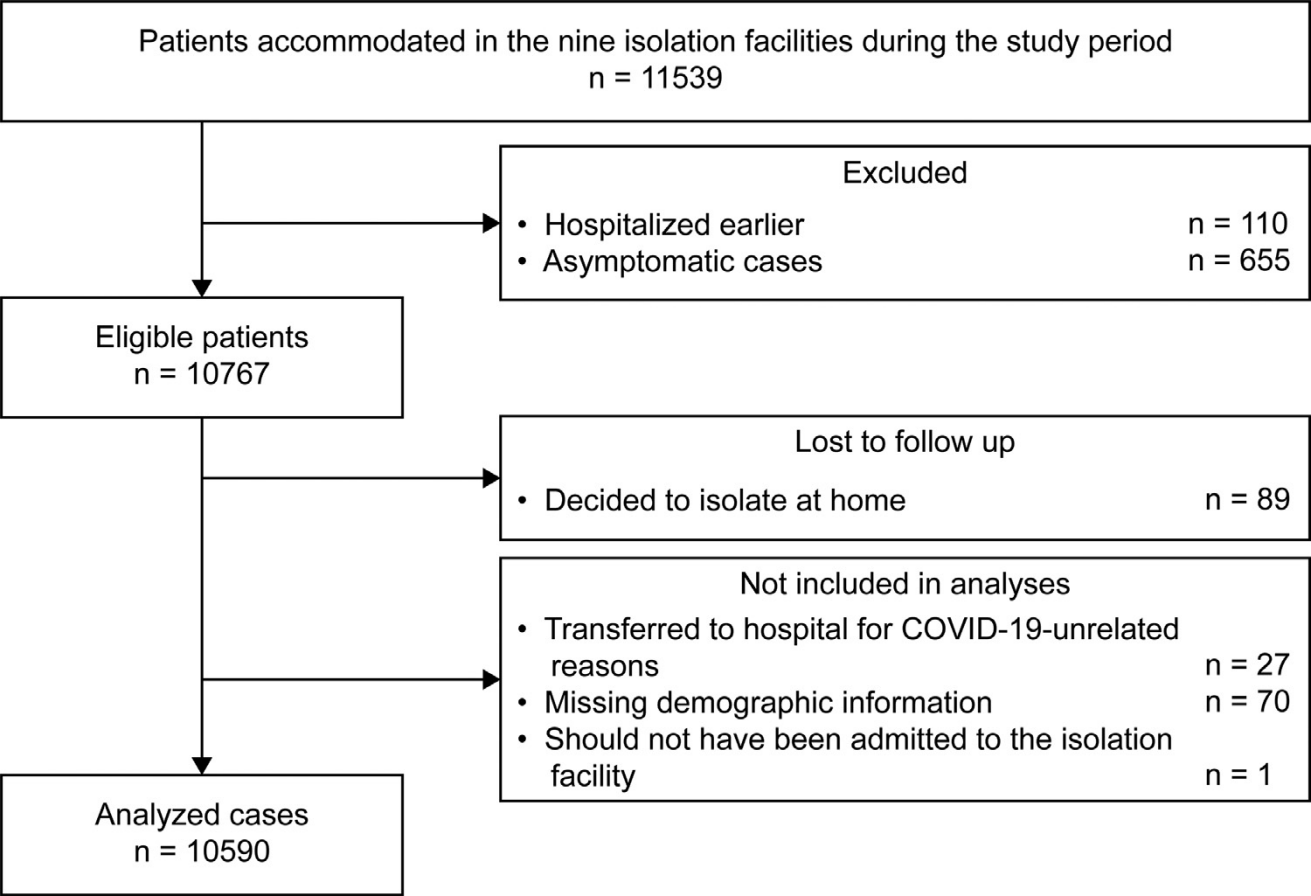
Cohort flow diagram. Patients admitted to and discharged from isolation facilities between November 1, 2020 and January 31, 2021 were included in the study.

In total, 11 539 patients were admitted to and discharged from isolation facilities during the study period (Figure 2). Of these, 655 (5.7%) were asymptomatic, and 110 (1.0%) were hospitalized and treated for COVID-19 prior to admission to the isolation facilities. Of the 10 767 eligible patients, 89 (0.8%) opted for self-isolation at home; almost none of these had any abnormal vital signs, and only four (4.5%) of these were febrile (Supplementary Table 1). Some patients were transferred to a hospital for reasons unrelated to COVID-19 (*n* = 27), two of which were later readmitted to the isolation facilities. Furthermore, 70 patients (0.7%) were excluded due to missing demographic information, and one due to deviation from the admission criteria.

In total, 10 590 patients were analyzed, of whom 10 223 (96.5%) safely completed the isolation period without major complications. The remaining 367 (3.5%) patients were eventually transferred to the hospital due to worsening COVID-19 symptoms, and 44 (12.0%) of these required an ambulance for urgent oxygen administration.

**Figure 3:**
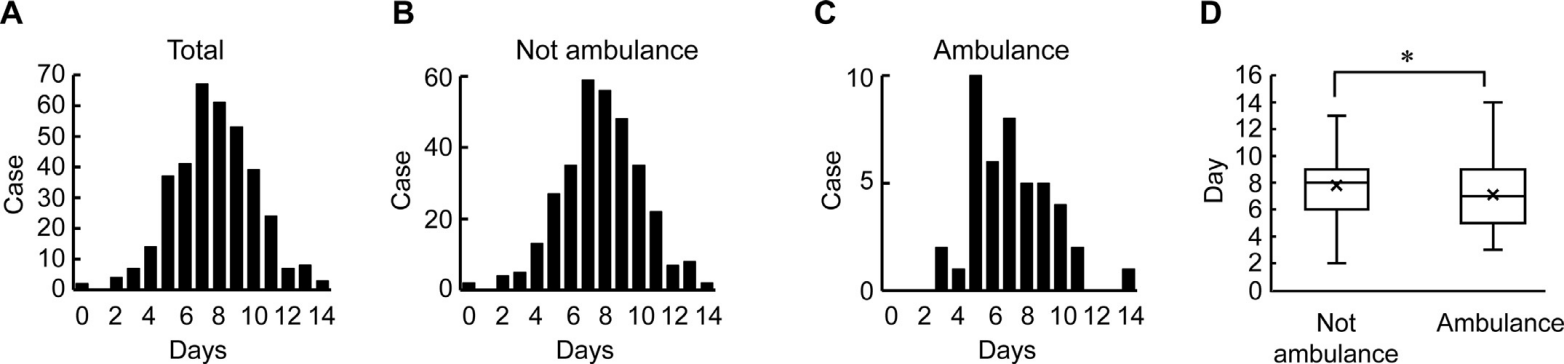
Days between symptom onset and hospital transfer from isolation facilities among patients with worsening COVID-19 symptoms. **(A)** Total cases (*n* = 367, 8 d (Chen et al., 2020b, Chia et al., 2021, Dickens et al., 2020, Docherty et al., 2020)). **(B)** Patients not transferred by ambulance (*n* = 323, 8 d [6-9]). **(C)** Patients transferred by ambulance (*n* = 44, 7 d (Chen et al., 2020a, Chen et al., 2020b, Chia et al., 2021, Dickens et al., 2020, Docherty et al., 2020)). Emergency transfer by ambulance occurred most frequently on day 5 (*n* = 10, 22.7%), whereas other transfers occurred most frequently on day 7 (*n* = 59, 18.3%). **(D)** Box plot comparing the data shown in Figures 3B and 3C (non-ambulance vs ambulance transfers). *p < 0.05.

The median duration from symptom onset to facility admission was 5 days (IQR: 4–6). Of the 367 transferred patients, the median duration from symptom onset to hospital transfer was 8 days (IQR: 6–9; range: 0–14) (Figure 3A). Patients transferred by ambulance owing to the rapid progression of symptoms experienced symptom worsening 1 or 2 days earlier than those who were not transferred (Figures 3B–3D).

Patient demographic data are presented in Table 1. The median age was 34 years (25–48). Over half of the participants were men (*n* = 6 475; 61.1%). Hypertension was the most common comorbidity (*n* = 704; 6.6%). The numbers of patients with chronic cardiovascular disease, diabetes, and respiratory diseases other than asthma were 82 (0.8%), 195 (1.8%), and 53 (0.5%), respectively. Older age, male sex, higher BMI, and several comorbidities, including hypertension, chronic cardiovascular disease, diabetes, and respiratory disease, were associated with an increased risk of hospital transfer based on univariate analysis results (Table 2).

**Table 1:** Demographic characteristics of study participants and results of the primary outcome univariate analysis

Variables^a^	Subcategories	Total(*N* = 10 590)	Transfer to hospital(*n* = 367)	isolation completed(*n* = 10223)	OR^b^ (95% CI^c^)(unadjusted)	*p*-value
Sex	Male	6475 (61.1)	284 (77.4)	6191 (60.6)	2.23 (1.73–2.89)	<0.001
	Female	4115 (38.9)	83 (22.6)	4032 (39.4)	Reference	
Age, median [IQR^d^], years		34 [25, 48]	54 [46, 60]	33 [25, 47]	–	< 0.001
Age group, years	0–9	25 (0.2)	0 (0)	25 (0.2)	0.00 (0.00–Inf)	0.97
	10–19	685 (6.5)	3 (0.8)	682 (6.7)	0.34 (0.10–1.13)	0.078
	20–29	3526 (33.3)	14 (3.8)	3512 (34.4)	0.31 (0.16–0.59)	< 0.001
	30–39	2118 (20.0)	27 (7.4)	2091 (20.5)	Reference	
	40–49	1940 (18.3)	84 (22.9)	1856 (18.2)	3.51 (2.26–5.43)	< 0.001
	50–59	1712 (16.2)	145 (39.5)	1567 (15.3)	7.17 (4.73–10.90)	< 0.001
	60–69	584 (5.5)	94 (25.6)	490 (4.8)	14.90 (9.58–23.00)	< 0.001
BMI, median [IQR], kg/m^2^		22.2 [20.1, 24.9]	25.1 [22.7, 27.6]	22.1 [20.1, 24.8]	–	< 0.001
BMI group, kg/m^2^	<25	8039 (75.9)	182 (49.6)	7857 (76.9)	Reference	
	≥25, <30	2004 (18.9)	138 (37.6)	1866 (18.3)	3.19 (2.54–4.01)	< 0.001
	≥30	547 (5.2)	47 (12.8)	500 (4.9)	4.06 (2.91–5.66)	< 0.001
Nationality	Japanese	10088 (95.3)	354 (96.5)	9734 (95.2)	Reference	
	Non-Japanese	502 (4.7)	13 (3.5)	489 (4.8)	0.73 (0.38–1.28)	0.32
Comorbidities	Hypertension	704 (6.6)	79 (21.5)	625 (6.1)	4.21 (3.20–5.50)	< 0.001
	Cardiovascular disease	82 (0.8)	11 (3.0)	71 (0.7)	4.42 (2.09–8.48)	< 0.001
	Bronchial asthma	305 (2.9)	28 (7.6)	277 (2.7)	2.97 (1.91–4.46)	< 0.001
	Obstructive sleep apnea	27 (0.3)	7 (1.9)	20 (0.2)	9.91 (3.52–24.61)	< 0.001
	COPD^e^ or emphysema	8 (0.1)	3 (0.8)	5 (0.0)	16.83 (2.60–87.01)	< 0.01
	Respiratory disease (other than asthma)	53 (0.5)	10 (2.7)	43 (0.4)	6.63 (2.95–13.54)	< 0.001
	Diabetes	195 (1.8)	39 (10.6)	156 (1.5)	7.67 (5.17–11.17)	< 0.001
	Dyslipidemia	449 (4.2)	48 (13.1)	401 (3.9)	3.69 (2.62–5.10)	< 0.001
	Hyperuricemia	293 (2.8)	39 (10.6)	254 (2.5)	4.67 (3.18–6.69)	< 0.001
	Chronic kidney disease	21 (0.2)	1 (0.3)	20 (0.2)	1.39 (0.03–8.76)	0.52
	Liver disease	66 (0.6)	9 (2.5)	57 (0.6)	4.48 (1.94–9.21)	< 0.001
	Recent use of immunosuppressants	15 (0.1)	0 (0)	15 (0.1)	0 (0.00–7.79)	1
	Stroke	26 (0.2)	5 (1.4)	21 (0.2)	6.71 (1.96–18.41)	< 0.01
	Malignancy	111 (1.0)	11 (3.0)	100 (1.0)	3.13 (1.50–5.91)	< 0.01
	Collagen disease	24 (0.2)	2 (0.5)	22 (0.2)	2.54 (0.29–10.40)	0.20
	Inflammatory bowel disease	42 (0.4)	5 (1.4)	37 (0.4)	3.80 (1.16–9.78)	< 0.05
	HIV^f^	13 (0.1)	1 (0.3)	12 (0.1)	2.32 (0.05–15.79)	0.37

a Data are presented as *n* (%), unless designated otherwise

b Odds ratio

c Confidence interval

d Interquartile range

e Chronic obstructive pulmonary disease

f Human immunodeficiency virus

**Table 2:** Results of univariate and multivariate analyses of the risks of hospital transfer

Variables^a^	Subcategories	Unadjusted OR(95% CI)	*p*-value	Adjusted OR^b^(95% CI^c^)	*p*-value
Age group, years	0–39	1 ()		1 ()	
	40–49	6.49 (4.49–9.38)	< 0.001	5.00 (3.44–7.27)	< 0.001
	50–59	13.30 (9.43–18.70)	< 0.001	11.00 (7.71–15.70)	< 0.001
	60–69	27.50 (19.00–39.80)	< 0.001	23.90 (16.20–35.50)	< 0.001
Male (vs. female)		2.23 (1.73–2.89)	< 0.001	1.84 (1.41–2.40)	< 0.001
BMI, kg/m^2^		–	–	1.10 (1.07–1.13)	< 0.001
Comorbidities	Hypertension	4.21 (3.20–5.50)	< 0.001	0.83 (0.60–1.13)	0.23
	Cardiovascular disease	4.42 (2.09–8.48)	< 0.001	1.09 (0.53–2.25)	0.81
	Bronchial asthma	2.97 (1.91–4.46)	< 0.001	2.17 (1.40–3.38)	< 0.001
	Respiratory disease (other than asthma)	6.63 (2.95–13.54)	< 0.001	1.90 (0.87–4.13)	0.11
	Diabetes	7.67 (5.17–11.17)	< 0.001	2.00 (1.32–3.03)	< 0.01
	Dyslipidemia	3.69 (2.62–5.10)	< 0.001	0.90 (0.62–1.29)	0.55
	Hyperuricemia	4.67 (3.18–6.69)	< 0.001	1.34 (0.90–1.99)	0.15
	Inflammatory bowel disease	3.80 (1.16–9.78)	< 0.05	3.18 (1.13–8.92)	< 0.05
	Liver disease	4.48 (1.94–9.21)	< 0.001	1.19 (0.54–2.65)	0.66
	Malignancy	3.13 (1.50–5.91)	< 0.01	1.42 (0.73–2.79)	0.30
	Stroke	6.71 (1.96–18.41)	< 0.01	2.61 (0.88–7.72)	0.084

a Data are presented as *n* (%), unless designated otherwise

b Odds ratio

c Confidence interval

After adjusting for confounders in the multivariate analysis, older age was found to be strongly associated with hospital transfer (adjusted odds ratio [AOR] = 23.90 and 95% CI [confidence interval] 16.20–35.50 for 60–69 years; AOR = 11.00 and 95% CI 7.71–15.70 for 50–59 years; AOR = 5.00 and 95% CI 3.44–7.27 for 40–49 years). Male sex (AOR = 1.84, 95% CI 1.41–2.40), BMI (AOR = 1.10, 95% CI 1.07–1.13), diabetes (AOR = 2.00, 95% CI 1.32–3.03), bronchial asthma (AOR = 2.17, 95% CI 1.40–3.38), and inflammatory bowel disease (AOR = 3.18, 95% CI 1.13–8.92) were also significantly associated with hospital transfer (Table 2). Among the 42 patients with inflammatory bowel disease, six (14.2%) had Crohn's disease, whereas the remaining 36 (85.7%) had ulcerative colitis. Only two of the 42 patients were treated with immunosuppressive agents (biologics and immunosuppressants), and all five transferred patients had ulcerative colitis.

Table 3 shows the demographic characteristics and comorbidities of patients transferred to hospital by ambulance in comparison with the control group. As with the primary outcome, patients requiring ambulance transport and oxygen administration were more likely to be older. The median age was 58 years (50.75–62.25). Male sex (*n* = 35, 79.5%) and higher BMI (25.2 [23.9–27.3] kg/m^2^) were significantly associated with an increased risk of ambulance transfer in the univariate analysis. Because of the limited number of cases, certain conditions, such as chronic kidney and liver disease, were not represented, making it difficult to accurately evaluate the risks of some comorbidities. Nevertheless, hypertension and diabetes were relatively prevalent among patients transferred by ambulance (*n* = 12 and 5, respectively), and were significantly associated with an increased risk of ambulance transfer (OR = 5.76, 95% CI 2.69–11.55 and OR = 8.27, 95% CI 2.51–21.39, respectively).

**Table 3:** Demographic characteristics of study participants and results of the secondary outcome univariate analysis

Variables^a^	Subcategories	Ambulance transfer (*n* = 44)	Isolation completed(*n* = 10 223)	OR^b^ (95% CI^c^)(unadjusted)	*p*-value
Sex	Male	35 (79.5)	6191 (60.6)	2.53 (1.19–6.00)	0.012
	Female	9 (20.5)	4032 (39.4)	Reference	
Age, median [IQR^d^], years		58 [50.75, 62.25]	33 [25, 47]	–	< 0.001
Age group, years	0–9	0 (0)	25 (0.2)	0.00 (0.00–Inf)	1
	10–19	0 (0)	682 (6.7)	0.00 (0.00–Inf)	0.98
	20–29	1 (2.3)	3512 (34.4)	0.20 (0.02–1.91)	0.16
	30–39	3 (6.8)	2091 (20.5)	Reference	
	40–49	6 (13.6)	1856 (18.2)	2.25 (0.56–9.02)	0.25
	50–59	17 (38.6)	1567 (15.3)	7.56 (2.21–25.80)	< 0.01
	60–69	17 (38.6)	490 (4.8)	24.20 (7.06–82.80)	< 0.001
BMI, median [IQR], kg/m^2^		25.2 [23.9, 27.3]	22.1 [20.1, 24.8]	–	< 0.001
BMI group, kg/m^2^	< 25	20 (45.5)	7857 (76.9)	Reference	
	≥ 25, < 30	18 (40.9)	1866 (18.3)	3.79 (2.00–7.18)	< 0.001
	≥ 30	6 (13.6)	500 (4.9)	4.71 (1.88–11.80)	< 0.001
Nationality	Japanese	41 (93.2)	9734 (95.2)	Reference	
	Non-Japanese	3 (6.8)	489 (4.8)	1.46 (0.29–4.59)	0.47
Comorbidities	Hypertension	12 (27.3)	625 (6.1)	5.76 (2.69–11.55)	< 0.001
	Cardiovascular diseases	1 (2.3)	71 (0.7)	3.32 (0.08–20.13)	0.27
	Bronchial asthma	1 (2.3)	277 (2.7)	0.84 (0.02–4.95)	1
	Obstructive sleep apnea	1 (2.3)	20 (0.2)	11.85 (0.28–77.71)	0.086
	COPD^e^ or emphysema	0 (0)	5 (0.0)	0.00 (0.00–259.43)	1
	Respiratory disease (other than asthma)	1 (2.3)	43 (0.4)	5.50 (0.13–33.95)	0.17
	Diabetes	5 (11.4)	156 (1.5)	8.27 (2.51–21.39)	< 0.001
	Dyslipidemia	2 (4.5)	401 (3.9)	1.17 (0.14–4.51)	0.69
	Hyperuricemia	3 (6.8)	254 (2.5)	2.87 (0.57–9.09)	0.097
	Chronic kidney disease	0 (0)	20 (0.2)	0.00 (0.00–48.76)	1
	Liver disease	0 (0)	57 (0.6)	0.00 (0.00–16.08)	1
	Recent use of immunosuppressants	0 (0)	15 (0.1)	0.00 (0.00–67.01)	1
	Stroke	1 (2.3)	21 (0.2)	11.29 (0.27–73.51)	0.090
	Malignancy	2 (4.5)	100 (1.0)	4.92 (0.56–18.97)	0.071
	Collagen disease	0 (0)	22 (0.2)	0.00 (0.00–43.96)	1
	Inflammatory bowel disease	0 (0)	37 (0.4)	0.00 (0.00–25.25)	1
	HIV^f^	1 (2.3)	12 (0.1)	19.76 (0.45–139.04)	0.054

a Data are presented as *n* (%), unless designated otherwise

b Odds ratio

c Confidence interval

d Interquartile range

e Chronic obstructive pulmonary disease

f Human immunodeficiency virus

Multivariate analysis was conducted to assess the effects of selected variables, including age, sex, and BMI, on ambulance transfer. These were all found to be significant risk factors for ambulance transfer (AOR = 8.23, 95% CI 3.75–18.10 for 50–59 years; AOR = 28.90, 95% CI 13.10–63.80 for 60–69 years; AOR = 2.32; 95% CI 1.10–4.90 for male sex; and AOR = 1.14, 95% CI 1.07–1.21 for BMI) (Table 4).

**Table 4:** Univariate and multivariate analyses of the risks of ambulance transfer

Variables^a^	Subcategories	Univariable OR (95% CI)	*p*-value	Multivariable OR^b^ (95% CI^c^)	*p*-value
Age group, years	0–49	1 ()		1 ()	
	50–59	8.86 (4.05–19.40)	< 0.001	8.23 (3.75–18.10)	< 0.001
	60–69	28.30 (12.90–62.20)	< 0.001	28.90 (13.10–63.80)	< 0.001
Male (vs female)		2.53 (1.19–6.00)	< 0.05	2.32 (1.10–4.90)	< 0.05
BMI, kg/m^2^		–	–	1.14 (1.07–1.21)	< 0.001

a Data are presented as *n* (%), unless designated otherwise

b Odds ratio

c Confidence interval

## Discussion

The risk factors associated with hospital transfer from isolation facilities in Tokyo among initially asymptomatic or mild COVID-19 patients were evaluated. After adjusting for confounders, older age, male sex, higher BMI, and comorbidities, including diabetes, bronchial asthma, and inflammatory bowel disease, were found to be significant risk factors for hospital transfer. Moreover, older age, male sex, and higher BMI were significant risk factors for hospital transfer by ambulance, indicating an urgent need for oxygenation.

### Comparisons with other studies in the literature

Our study cohort was unique, owing to the admission criteria of the isolation facilities, i.e. an age limit of 69 years and well-managed comorbidities. For instance, patients with a regular systolic blood pressure of > 180 mmHg or HbA1c levels > 10% were rarely admitted. Nevertheless, our results were in agreement with the findings of previous studies, which showed that older age, male sex, obesity, and diabetes were risk factors for severe COVID-19 infection (Matsunaga et al., 2020; Popkin et al., 2020; Zheng et al., 2020; Yang et al., 2020; Fadini et al., 2020; Tian et al., 2020; Yang et al., 2021). A meta-analysis that included 14 studies and 4659 patients reported that several factors, including male sex (OR = 1.78, 95% CI 1.30–2.42), cardiovascular disease including coronary artery disease (OR = 3.81, 95% CI 2.11–6.85), and diabetes (OR = 1.97, 95% CI 1.67–2.31), increased mortality (Tian et al., 2020). Another meta-analysis revealed that higher BMI contributed to a more severe COVID-19 infection, in which the weighted mean BMI deviation between severe and non-severe patients was 2.67 kg/m^2^ (95% CI 1.52–3.82) (Yang et al., 2021). Our data highlight the importance of these predisposing factors for severe COVID-19 infection that requires medical attention.

However, our results for some comorbidities, such as hypertension, cardiovascular disease, and chronic respiratory disease other than bronchial asthma, were not significant, although they had been previously identified as risk factors (Zheng et al., 2020; Lippi and Henry, 2020; Tian et al., 2020; Huang et al., 2020; Docherty et al., 2020; Williamson et al., 2020). For example, a meta-analysis that included 13 publications showed that hypertension, cardiovascular diseases, and respiratory diseases were more prevalent in critical and fatal cases of COVID-19 than in non-critical cases (OR = 2.72, 95% CI 1.60–4.64; OR = 5.19, 95% CI 3.25–8.29; and OR = 5.15, 95% CI 2.51–10.57, respectively) (Zheng et al., 2020). Aside from the definitions of the outcomes, our results suggest that these discrepancies are partly due to selection bias, as we did not include patients who were expected to develop moderate to severe symptoms on the basis of previous evidence. The low prevalence of hypertension in our cohort compared with that in a previous study highlighted this bias (Matsunaga et al., 2020). The resulting small number of patients with comorbidities, such as cardiovascular and chronic respiratory disease, restricted the statistical power, possibly affecting the results of the analysis.

Compared with other respiratory diseases, bronchial asthma has not been established as a risk factor for severe COVID-19; however, theoretically, these patients can be at a higher risk owing to a deficient antiviral immune response and exacerbation of bronchial asthma following viral infection (Liu et al., 2020; Gao et al., 2021; Zhu et al., 2020). A large-scale cohort study conducted in the UK reported that asthma was associated with severe COVID-19 (AOR = 1.39, 95% CI 1.13–1.71) (Zhu et al., 2020). Our results suggest that underlying bronchial asthma can be a potential risk factor for COVID-19 patients who require medical care. Further investigation should focus on other factors, such as treatment regimen, severity of asthma, and disease control status (Gao et al., 2021; Zhu et al., 2020).

Whether inflammatory bowel disease is associated with an increased risk of severe COVID-19 remains unclear (Sultan et al., 2020). One observational study in Spain revealed that of the 40 reported COVID-19 patients with inflammatory bowel disease, 21 (53%) were hospitalized and two (5%) died due to complications with acute respiratory distress syndrome (Rodríguez-Lago et al., 2020). In another prospective observational study in Italy, a diagnosis of ulcerative colitis was significantly associated with COVID-19 pneumonia compared with that of Crohn's disease (OR = 2.72, 95% CI 1.06–6.99) and disease activity (OR = 10.25, 95% CI 2.11–49.73) (Bezzio et al., 2020). Interestingly, all five patients with inflammatory bowel disease in our cohort had ulcerative colitis. However, our results should be interpreted with caution as the number of patients who had these diseases was small. Among the 42 patients with inflammatory bowel disease in this study, only two were treated with immunosuppressive agents. As these patients safely completed the isolation period, the possibility that our results were due to treatment side-effects was excluded (Brenner et al., 2020; Bezzio et al., 2020).

### Patients transferred by ambulance and silent hypoxemia

Because the symptoms of patients who require ambulance transport can progress rapidly, a delayed response could result in serious consequences. Therefore, it is essential to identify the patients at risk. Our results support this idea because patients transferred by ambulance experienced deterioration 1 or 2 days earlier than those who were not. However, because the number of patients examined in this study was small, most comorbidities could not be evaluated by multivariate analysis. Nevertheless, hypertension and diabetes were identified as possible risk factors by univariate analysis.

Some patients transferred by ambulance presented with silent hypoxemia (Tobin et al., 2020; Quaresima and Ferrari, 2020), i.e. having a low SpO_2_ level (< 90%) in the absence of dyspnea (or presence of mild dyspnea). Therefore, merely relying on patients’ complaints may not be sufficient. Although SpO_2_ is a less accurate measure than SaO_2_ (Tobin et al., 2020), its routine monitoring using pulse oximeters helps to avoid overlooking such cases, regardless of patient symptoms.

### Limitations of the study

This study had several limitations. Firstly, patients with considerable risk of worsening COVID-19 prior to admission to the isolation facilities were excluded. Secondly, our study relied on self-reported data regarding underlying diseases and medications. Therefore, there were considerable limitations regarding the accuracy and comprehensiveness of such information, partly due to recall bias and noncooperation. Similarly, non-Japanese patients might have underreported their underlying medical issues because of language barriers. Thirdly, only a limited number of patients underwent hospital transfer, especially by ambulance. Finally, data on the outcomes for transferred and hospitalized patients in terms of admission to the intensive care unit, ventilator use, or death were not collected.

## Conclusion

The isolation facilities of the Tokyo Metropolitan Government have provided patients with an accommodating environment wherein they are monitored by medical providers and participate in saving resources. Our study found that age, male sex, higher BMI, and several comorbidities, such as diabetes, bronchial asthma, and inflammatory bowel disease, were risk factors associated with hospital transfer from isolation facilities. Patients with more severe infections who required urgent oxygen administration tended to progress more rapidly, and routine SpO_2_ monitoring was a useful method to detect these cases. Identifying these risk factors provides practical benefits in developing preparation measures for future waves of COVID-19 infection. Future studies should investigate admissions to the intensive care unit, ventilator use, and deaths as outcomes to ensure patient safety and the more efficient use of medical resources.

## Declaration of Competing Interests

The authors declare that they have no known competing financial interests or personal relationships that could have influenced the work reported in this paper.

## Appendix Supplementary materials

Image, application 1
