# Supplementary material for: Risk factors associated with hospital transfer among mild or asymptomatic COVID-19 patients in isolation facilities in Tokyo: a case-control study
Source: IJID Reg. 2021 Nov 17;2:8–15. doi: 10.1016/j.ijregi.2021.11.001 (PMC8595257; doi:10.1016/j.ijregi.2021.11.001)
Supplement: Supplementary file 1 [file mmc1.docx]

**Supplementary Table 1.** Reasons for switching from isolation at the facilities to home as well as the prevalence of abnormal vital signs.

**(A)** The most frequent reason was environmental stress including claustrophobia, possibly due to restrictions against moving freely outside the room. The second most frequent reason was family-related. For example, the partner of the patient being accommodated also got infected with COVID-19 or was hospitalized leaving their children at home.

| **Reasons for isolating at home** | **Total (n = 89)**  **n (%)** |
| --- | --- |
| Environmental stress including claustrophobia | 41 (46.1) |
| Family-related | 28 (31.5) |
| Non-consent | 6 (6.7) |
| Comorbidities | 6 (6.7) |
| Other or unknown | 8 (9.0) |

**(B)** Most patients presented without any abnormal vital signs but only four (4.5%) were febrile. SpO_2_ <96% was not observed.

| **Vital sign abnormalities upon switching from facility to home isolation** | **Total (n = 89)**  **n (%)** |
| --- | --- |
| Febrile (>37.5˚C) | 4 (4.5) |
| SpO_2_ <96% | 0 (0) |
